# Venous Thromboembolism Prophylaxis after Hematopoietic Cell Transplantation: Still a Challenge for Hematologists and Hemostasiologists

**DOI:** 10.1055/a-2434-5010

**Published:** 2024-11-11

**Authors:** Paola Ranalli, Hugo ten Cate

**Affiliations:** 1 Hematology Unit, Pescara Hospital, Pescara, Italy; 2 Department of Medicine and Aging Sciences, University of Chieti-Pescara, Chieti, Italy; 3 Department of Internal Medicine and Thrombosis Expert Center, Maastricht University Medical Centre, and CARIM School for Cardiovascular Diseases, Maastricht, The Netherlands; 4 Center for Thrombosis and Hemostasis, Gutenberg University Medical Center, Mainz, Germany.

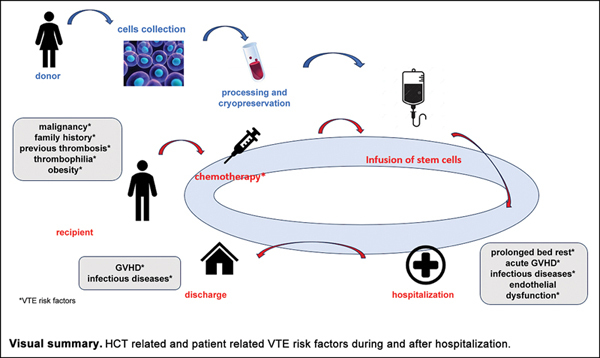

Venous Thromboembolism Post-allogeneic Hematopoietic Cell Transplant: Risk Factors, Incidence, and Outcomes

Allogeneic hematopoietic cell transplantation (HCT) represents the only curative option for several hematological malignancies. As a consequence of improved conditions (donor selection, stem cell sources, supportive care, prevention of complications, and reduced-toxicity preparative regimens), also including better collaboration among centres, HCT can be performed in almost all patients with a specific indication. 1 Furthermore, it is now also routinely and safely performed in elderly and in patients with comorbidities. For all these reasons its application has significantly expanded over the last years. 2

Beyond relapse, still considered the predominant cause of failure, HCT remains associated with relevant morbidity and mortality. In comparison with other clinical complications, morbidity and mortality related to thromboembolism is much less explored in this setting. Perhaps for that reason, previous thromboembolism is not listed among comorbidities included in the Hematopoietic Cell Transplantation Comorbidity Index, the most often used score to predict survival after transplantation. 3

In the study by Granat et al 4 published in this issue of Thrombosis and Haemostasis, the authors report posttransplant thromboembolic complications in a retrospective cohort of 431 hematological patients, mainly affected by acute myeloid leukemia and myelodysplastic syndromes, who underwent bone marrow transplantation in the period 2014 to 2019 at the Cleveland Clinic Main Campus, Ohio, United States. Chronic myeloproliferative neoplasms, more prone to thrombosis development, accounted just for 5% of patients.

In their analysis the risk of venous thrombosis was higher than observed in a recent meta-analysis including patients in the same setting (14.8 vs. 4%, respectively). 5 Furthermore, venous thromboembolism (VTE) was significantly associated with increased risk of nonrelapse mortality and decreased survival. The risk of VTE after HCT remained high even a long time after the procedure; in this study VTE incidence continued to rise beyond 1 year, in contrast with other most feared complications like sepsis.

Isolated pulmonary embolism represented 12.5% of all cases of VTE in this study, which merits some discussion on an important differential diagnosis: in cases without evidence of deep venous thrombosis, local thrombotic microangiopathy, resulting from endothelial cell activation, orchestrating inflammatory and thrombotic responses, complement dysregulation, and microvascular hemolytic anemia, must be considered. In patients with pulmonary vascular involvement, microangiopathy can present as a respiratory distress syndrome, with hypoxemia related to pulmonary hypertension and potentially devastating consequences. Although not common after HCT, microangiopathy is included among HCT-related complications and must be excluded as its treatment relies on measures to limit endothelial injury (avoidance of endothelial toxins, preventing or treating infection, and optimization of conditioning regimens, including complement inhibition) and not simply on anticoagulation. 6

None of the patients that underwent HCT received primary thromboprophylaxis and no data are available about mechanical thromboprophylaxis in this cohort. Surprisingly, none of the patients who experienced thromboembolism after HCT were on prophylaxis at the time of VTE relapse. While the authors do not address the general avoidance of thromboprophylaxis in this population, one can think of several reasons. First, HCT per se is still not perceived as a condition with a high thrombotic risk, despite recognized risk factors for thrombosis in this setting. Second and probably relevant for many hemato-oncological conditions, there are general concerns about the risk of bleeding related to thrombocytopenia in HCT patients, 7 8 9 10 although the median platelet count at diagnosis of VTE in this cohort was 101 × 10 ^3^ /µL and despite studies showing VTE prophylaxis does not increase bleeding risk in HCT. 11 12 Additionally, some data argue against routine pharmacological VTE prophylaxis in patients undergoing HCT, despite insufficient evidence to support this recommendation. 13 Finally, there are concerns that routine low molecular weight heparin (LMWH) does not sufficiently protect patients from catheter-associated thrombosis in cancer 14 or may only have a modest impact on reducing the rate of VTE as suggested by a study in hospitalized HCT patients receiving subcutaneous LMWH or unfractionated heparin, as compared with a historic control population not receiving pharmacological prophylaxis, without increase of bleeding. 15

The point is: are clinicians sufficiently aware of the thromboembolic risk during recovery from HCT? A greater uptake of risk assessment tools that have been developed for patients undergoing HCT could improve VTE risk management, provided that these decision support methods are further improved and implemented. 16 17 18

One of the important thrombosis risk factors is graft-versus-host disease (GVHD). Systemic inflammation and endothelial dysfunction associated with chronic GVHD (cGVHD) increases the risk of thromboembolic as well as bleeding events after HCT, even in the long term. 19 A recent study reported a 22% 5-year cumulative incidence of thromboembolic events with a median time between cGVHD diagnosis and thrombosis of 234 days, becoming even longer for upper extremity DVT (median time: 450 days). Severe cGVHD, non-O donor–recipient and previous history of coronary artery disease were factors associated with higher risk. 20 In contrast with previous studies, in the study by Granat et al, 4 an association between GVHD and thromboembolism was found only for acute GVHD.

Regarding thrombosis management, Granat et al conclude that therapeutic dosed anticoagulation with LMWH or direct oral anticoagulants (DOAC) was generally safe, although significant bleeding complications still occurred in 9 out of 64 (14.1%) treated patients, 3 of whom had a major bleeding requiring intensive care support and placement of a caval filter, confirming data about safety of apixaban for thrombosis treatment in the Caravaggio trial 21 and ADAM VTE. 22

In this setting, the platelet count is the most relevant limiting factor for implementing any form of anticoagulation; nevertheless thrombocytopenia was not the main determinant of bleeding risk if we consider, as the authors state in the discussion, that bleeding patients had a higher platelet count at the time of VTE diagnosis. In line with a previous publication, 19 cGVHD was more frequent in bleeding patients than in nonbleeding patients (55.6 vs. 23.6%) in this study.

A certain scientific interest about the possibility of reducing VTE incidence, morbidity, and mortality in the setting of HCT is growing. The study by Granat et al represents a concrete example, offering several points of reflection for both hemostasiologists and hematologists.

Although we also recognize the complexities of thrombosis prevention in the HCT population, the overall incidence of VTE of 14% in this study, most of whom were not catheter-related (only 18.8% of all cases, which is different in other studies where catheter-related thrombosis is more prevalent), is such that any form of prophylaxis should be considered. 23 Given the emergence of specific risk factors like thrombocytopenia and GVHD in the course of HCT, one can imagine that physicians prefer to use a risk score in order to select patients for prophylaxis or to start with mechanical prophylaxis, if available. Even then, risk prediction models for VTE and VTE-related complications need developed for this population, but these will need to consider established risks for VTE and new methodologies, such as machine learning. 24

Either way, better safe than sorry, as the occurrence of VTE also indicates a higher mortality risk (although not necessarily causally linked). When VTE occurs, immediate treatment should commence following bleeding risk assessment, also considering important determinants of bleeding related to renal function and potential drug–drug interactions for DOACs. 25 26
